# The Association Between Religiosity, Spirituality, and Medication Adherence Among Patients with Cardiovascular Diseases: A Systematic Review of the Literature

**DOI:** 10.1007/s10943-022-01525-5

**Published:** 2022-03-10

**Authors:** Marwa Elhag, Ahmed Awaisu, Harold G. Koenig, Mohamed Izham Mohamed Ibrahim

**Affiliations:** 1 College of Pharmacy, Omdurman Islamic University, Omdurman, Sudan; 2 Department of Clinical Pharmacy and Practice, College of Pharmacy, QU Health, Qatar University, Doha, Qatar; 3 Department of Psychiatry and Behavioral Sciences and Department of Medicine, Duke University Medical Center, Durham, NC USA

**Keywords:** Religiosity, Spirituality, Medication adherence, Heart failure, Hypertension, Cardiovascular disease

## Abstract

This systematic review aimed to summarize the literature on the relationship between religiosity or spirituality (R/S) and medication adherence among patients with cardiovascular diseases (CVDs) and to describe the nature and extent of the studies evaluating this relationship. Seven electronic databases (PubMed, MEDLINE, EMBASE, Scopus, the Cochrane Central Library, ProQuest Theses and Dissertations, and Google Scholar) were searched with no restriction on the year of publication. The Crowe Critical Appraisal Tool was used to evaluate the methodological quality of the eligible studies. Due to the heterogeneity observed across the included studies, data synthesis was performed using a narrative approach. Nine original studies published between 2006 and 2018 were included in the review. Only a few quantitative studies have examined the relationship between R/S and medication adherence among patients with CVDs. Most studies were conducted in the USA (*n* = 7) and involved patients with hypertension (*n* = 6). Five studies showed a significant correlation between R/S (higher organizational religiousness, prayer, spirituality) and medication adherence and revealed that medication adherence improved with high R/S. The other four studies reported a negative or null association between R/S and medication adherence. Some of these studies have found relationships between R/S and medication adherence in hypertension and heart failure patients. This review showed a paucity of literature exploring the relationship between R/S and medication adherence among patients with other CVDs, such as coronary artery diseases, arrhythmia, angina and myocardial infarction. Therefore, the findings suggest that future studies are needed to explore the relationship between R/S and medication adherence among patients with other types of CVDs. Moreover, there is a need to develop interventions to improve patients’ medication-taking behaviors that are tailored to their cultural beliefs and R/S.

## Introduction

Religiosity is an important determinant of societal health (Zimmer et al., 2019). According to Zimmer et al. (2019), the relationship between health and religiosity is complex. Religiosity can influence the health behaviors of individuals with cardiovascular diseases (CVDs), such as coronary heart disease, hypertension and cerebrovascular disorders (Koenig, 2012). CVDs are non-communicable chronic disorders that are highly prevalent globally and are the leading cause of morbidity and mortality worldwide. The number of annual deaths due to CVDs is approximately 17.9 million. In addition, CVDs are associated with polypharmacy and medication non-adherence. Approximately 50% of patients suffering from these conditions are non-adherent to their prescribed medications (World Health Organization, 2018). For example, patients with heart failure can have poor adherence to their medications, which eventually leads to increased hospitalization and mortality (Hope et al., 2004; McMurray & Stewart, 2000). Thus, patient education, counseling, and effective communication between health care providers and patients are fundamental to improving health outcomes.

According to the World Health Organization (WHO), adherence can be defined as “the degree to which the person's behavior corresponds with the agreed recommendations from a health care provider” (Sabaté, 2003). Medication non-adherence in patients with CVDs leads to increased morbidity and mortality and decreased quality of life (Shehab et al., 2016). Conversely, medication adherence plays a crucial role in enhancing health-related quality of life, especially in patients with heart failure (Huang et al., 2018). Patients with chronic diseases who are prescribed multiple medications are usually burdened with a large amount of health information to process and understand, including the importance of taking the medications (Fredericksen et al., 2018). This problem is widespread in CVD patients since they are often exposed to multiple medications. As a result, poor adherence is widely prevalent, resulting in the poor control of important risk factors, such as blood pressure and cholesterol levels, and ultimately poor treatment outcomes (Kronish & Ye, 2013). Medication adherence is affected by many factors, including but not limited to health literacy, polypharmacy, cognitive function, and adverse drug events (Gellad et al., 2011). In addition, the published literature has documented that medication adherence is affected by religious and cultural beliefs (Lin et al., 2018). However, studies investigating the association between religiosity and health outcomes, including medication-taking behaviors and adherence, are scarce.

Interest in the association between religiosity and health has increased in recent years. Some studies have shown a positive relationship between religiosity and health outcomes (Kiecolt-Glaser et al., 2002; Larson et al., 1989; Plante & Sharma, 2001). The interaction between religion and health has attracted many researchers to investigate the effect of religion on health. For example, Levin and Vanderpool discussed the mechanism of religion in reducing blood pressure. They found that religious practices such as prayer and meditation could induce relaxation, reduce sympathetic nervous system activity, and decrease heart rate and blood pressure (Vanderpool & Levin, 1990).

In general, a patient prefers to be treated as a whole person rather than someone suffering from a disease. “A whole person is someone who’s being has physical, emotional, and spiritual dimensions” (Koenig, 2000). Thus, neglecting these facets can make patients feel dissatisfied with their care and ultimately affect their healing process (Koenig, 2000). Consequently, the American College of Physicians recognizes the role of religiosity/spirituality (R/S) in improving health care outcomes. There are four questions suggested by the American College of Physicians that might help patients: (1) “Is faith (religion, spirituality) important to you in this illness?”; (2) “Has faith been important to you at other times in your life?”; (3) “Do you have someone to talk to about religious matters?”; and (4) “Would you like to explore religious matters with someone?” (Koenig, 2000). Both R and S are interchangeable and related in most social science research (Kiecolt-Glaser et al., 2002; Mattis, 2000). Therefore, R or S may be a beneficial topic for interventions, especially for patients with chronic diseases (Huang et al., 2018). In this review, we evaluated patients’ R and S and whether they can affect medication adherence.

Religiosity (R) has been defined as “an individual’s belief toward a divinity” (Yaghoobzadeh et al., 2018). More specifically, according to Koenig (2018), “religiosity involves beliefs, practices, and rituals related to the ‘transcendent,’ where the transcendent relates to the mystical, supernatural, or God in Western religious traditions, or to Brahman, the Ultimate Truth, the Ultimate Reality, or practices leading to enlightenment, in Eastern traditions.” Beliefs about spirits, angels, or demons are also related to religion. Koenig (2018) further mentioned that “generally, religion involves specific beliefs about life after death and rules to guide personal behaviors and interactions with others during this life.”

Religion is frequently organized and practiced within a community. However, religion can also be practiced alone and privately outside of an institution, such as personal beliefs about and the commitment to transcendent and private activities such as prayer, meditation, and scripture study. Therefore, the term religion is not limited to organized religion, religious affiliation or religious attendance. Central to its definition, however, is that religion is rooted in an established tradition that arises from a group of people with shared beliefs and practices concerning the “transcendent” (p. 13) (Koenig, 2018). Spirituality is similar to religion, even though it may be broader in some faith traditions extending beyond religion (comprising the outcomes of devout religious involvement, such as having importance and an aim in life or being intimately connected to the divine) (Koenig et al., 2012).

Hyman and Handal (2006) conducted a pilot study to clarify whether the concept of religion and that of spirituality are the same or different. Four different types of religious professionals (imams, ministers, priests and rabbis) participated in this study. The study showed that 37% of the participants agreed that “spirituality is a broader concept than religion and includes religion.” In comparison, 18% of the participants agreed that “religion is a broader concept than spirituality and includes spirituality.” Moreover, 28% of the participants said that “religion and spirituality overlap but they are not the same concept”; 17% agreed that “religion and spirituality are the same concept and overlap completely”; and no participants agreed with the concept that “religion and spirituality are different and do not overlap.” In general, religious professionals displayed similar variability, except those in the Islamic group, who agreed that religion and spirituality are the same concept and overlap completely (Hyman & Handal, 2006).

Previous studies have reported an association between R or S and CVDs (Kobayashi et al., 2015; Trevino & McConnell, 2014), indicating that a higher level of R/S leads to better CVD outcomes. One study reported a positive correlation between R/S, personal beliefs and medication adherence in outpatients with heart failure (Alvarez et al., 2016). A study in the USA found that spiritual counseling effectively controlled the complications of heart failure and improved the patients’ quality of life (Tadwalkar et al., 2014).

Several studies from different parts of the world have reported a relationship between religiosity and medication adherence among patients diagnosed with hypertension or heart failure (Abel & Greer, 2017; Alvarez et al., 2016; Black et al., 2006; Harvin, 2018; Kretchy et al., 2013; Loustalot, 2006; Park et al., 2008; Yon, 2013). However, there is conflicting evidence regarding the direction and nature of this relationship. Moreover, no comprehensive systematic review investigating the association between R or S and medication adherence among patients with CVDs has been previously published. A deeper understanding of this relationship will help design spiritually based interventions (e.g., spiritual counseling) to improve medication-taking behaviors and adherence among patients with these disorders. Hence, this systematic review was undertaken to summarize the literature on the relationship between R/S and medication adherence and to describe the nature and extent of (i.e., to characterize and quantify) the studies evaluating the relationship.

## Methods

This systematic review was conducted following the Preferred Reporting Item for Systematic Reviews and Meta-Analysis (PRISMA) guidelines (Moher et al., 2009).

### Eligibility Criteria

Studies were included if they measured both patients’ R/S and medication adherence. The study population consisted of patients with a range of CVDs, including but not restricted to heart failure, acute coronary syndrome, and hypertension. The review included patients of all ages and from all settings. We included only articles published in the English language. Quantitative observational studies, randomized controlled trials, and qualitative studies were included. Non-research articles or non-original research articles (e.g., editorials, reviews, and letters) were excluded from the review.

### Search Strategy

A systematic literature search was conducted to identify published studies investigating CVD patients’ R/S and medication adherence. We searched the following electronic databases for eligible studies with no limit on publication year (i.e., from inception to the search date): PubMed, MEDLINE, EMBASE, Scopus, the Cochrane Central Library, ProQuest Theses and Dissertations, and Google Scholar. Searches were conducted between August 17, 2018, and September 5, 2018. In addition, manual searches of the retrieved articles’ bibliographies were conducted to identify studies that were not found in the electronic searches. Two reviewers (ME and MIMI) used Medical Subject Heading (MeSH) terms and suitable keyword synonyms related to “religiosity” and “spirituality” (including the related terms of religiousness, religious beliefs, religion, and spiritual) in combination with “medication adherence” (including the related term and synonym “medication compliance”) and “cardiovascular diseases” (including the related terms of “heart diseases”, “heart failure”, “acute myocardial infarction”, “acute coronary syndrome”, and “hypertension”). Appendix 1 shows the details of the search strings. All search results were imported into EndNote® reference management software version 8.

### Study Selection Process

The included studies were first assessed by an initial review of the article title and an abstract review. Then, the full texts of the publications that met the inclusion criteria were retrieved for data extraction. Two investigators (ME and MIMI) independently conducted the study selection process and review. The reviewers met several times to discuss the study eligibility criteria. In the case of discrepancies, a third reviewer (AA) provided the final judgment regarding the inclusion or exclusion of the articles.

### Data Extraction

The reviewers developed and piloted a standardized data extraction form to obtain information relevant to this systematic review. The extraction form included data on the authors, publication year, country of study, setting and population, study design, sample size, type of CVD, religion type, R/S measure, medication adherence measure, major findings, study limitations, and conclusions. In addition, detailed information regarding the outcome measures (R/S and medication adherence) was obtained, including the number of items and the description of the tool and scoring systems. The two reviewers (ME and MIMI) extracted the data, and the third investigator (AA) provided the final judgment regarding data extraction.

### Quality Assessment

The authors used the Crowe Critical Appraisal Tool (CCAT) to analyze and assess the methodological quality of the included studies. The CCAT is suitable for all research designs (i.e., quantitative, qualitative, and mixed-method designs). The eight assessed categories are the preliminaries, introduction, design, sampling, data collection, ethical issues, results, and discussion. Each category is scored from 0 to 5, where a higher score indicates a higher quality (Crowe & Sheppard, 2011a, b). A quality assessment score was calculated as a percentage for each study according to the CCAT User Guide (Crowe et al., 2011). The quality assessment was performed by two independent reviewers (ME and MIMI). The percentage agreement between the reviewers was determined using Cohen’s kappa test.

### Data Synthesis

Meta-analysis could not be conducted in this systematic review due to the methodological heterogeneity of the predictor/outcome measures and the research designs across the included studies. However, according to the University of York criteria, a narrative approach to data synthesis is recommended in systemic reviews to arrive at the conclusions of studies that vary in design (Popay et al., 2006). Thus, a qualitative narrative synthesis was used to summarize the findings of this review.

## Results

### Study Selection

**Fig. 1:**
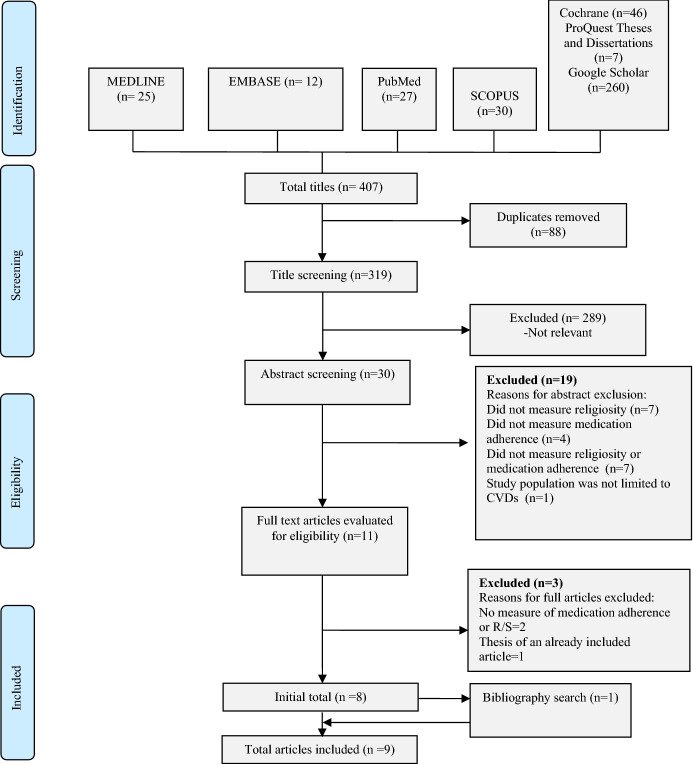
PRISMA flowchart of the included studies

Four hundred and seven references were identified from the electronic bibliographic database searches, from which 88 duplicates were removed. After screening the titles and abstracts, only 11 studies potentially met the eligibility criteria for inclusion; full-text versions of these studies were retrieved for review. Of these, two studies were excluded due to the omission of R/S and medication adherence measures. One study was excluded because it was a thesis based on a study that was already included in the review. One additional study was included based on the search of the included articles’ reference lists. As a result, nine studies (Abel & Greer, 2017; Alvarez et al., 2016; Black et al., 2006; Greer & Abel, 2017; Harvin, 2018; Kretchy et al., 2013; Loustalot, 2006; Park et al., 2008; Yon, 2013) were included in the present systematic review. A PRISMA flowchart describing the search results and selection process is provided in Fig. 1.

### Description Of Included Studies

#### Designs And Countries Of Study

The nine included studies were published between 2006 and 2018. Most studies (*n* = 7) were conducted in the USA (Abel & Greer, 2017; Black et al., 2006; Greer & Abel, 2017; Harvin, 2018; Loustalot, 2006; Park et al., 2008; Yon, 2013), while the others were performed in Ghana (Alvarez et al., 2016) and Brazil (Kretchy et al., 2013). Many of the studies (*n* = 6) used a cross-sectional design (Abel & Greer, 2017; Alvarez et al., 2016; Kretchy et al., 2013; Loustalot, 2006; Park et al., 2008; Yon, 2013); one study used a mixed-methods design (Greer & Abel, 2017); one utilized an experimental design (Harvin, 2018); and one used a cohort design (Black et al., 2006). The sample sizes of the studies ranged from 10 to 5302.

#### Participant Characteristics

The age of the study participants ranged from 19 to 85 years. The majority of participants were women in most studies. Two studies were conducted specifically among African American women (Abel & Greer, 2017; Greer & Abel, 2017), whereas three studies were conducted predominantly among males (Black et al., 2006; Greer & Abel, 2017; Park et al., 2008). In the studies performed in the USA, Brazil, and Ghana, the participants were primarily Christian, while a fourth study reported that approximately 90% of the participants were Christian, 5% were Muslim, and 1% identified with traditional religion (Kretchy et al., 2013). Three studies did not specify the religion of the participants (Alvarez et al., 2016; Harvin, 2018; Park et al., 2008). Six of the nine studies enrolled patients with hypertension (Abel & Greer, 2017; Greer & Abel, 2017; Harvin, 2018; Kretchy et al., 2013; Loustalot, 2006; Yon, 2013), while three studies involved patients with heart failure (Alvarez et al., 2016; Black et al., 2006; Park et al., 2008). A detailed description of the included studies is presented in Table 1.

**Table 1:** Summary of the included studies

Authors (publication year), country, (reference)	Study design/sample size	Cardiovascular disease type and setting	Religiosity and/or spirituality measure/religion(s) type	Medication adherence measure	Major findings	Limitation(s)	Conclusion(s)
Abel WM & Greer DB (2017), USA (W. M. Abel & D. B. Greer, 2017)	Cross-sectional study (*n* = 80)	Hypertension, North Carolina hair salons, Black churches, community events, and social nomination	1. Nine spiritual/religious questions developed by the investigator (Christianity)	One subscale of the 14-item Hill-Bone Compliance to High Blood Pressure Therapy scale	1. No significant correlation between medication adherence and attending church/religious services, praying, reading Bible/religious material, and strength of spiritual beliefs 2. Data support that as spiritual and religious beliefs increased, medication adherence increased, but this relationship did not reach statistical significance	1. The sample was primarily recruited by snowball technique through church members 2. Non-random and small sample size used in the research 3. Study results are limited by the low reliability of the new investigator-developed spiritual/religious data collection tool	There was no significant association between religious beliefs, spirituality and medication adherence among African American women suffering from hypertension
Alvarez et al. (2016), Brazil (Alvarez et al., 2016)	Cross-sectional study (*n* = 130)	Heart failure clinic at the Hospital de Clínicas de Porto Alegre	1. Duke University Religion Index (DUREI) 2. The World Health Organization Quality of Life Spirituality, Religiosity and Personal Beliefs (WHOQoL SRPB) (No religion reported)	Repetitive Education and Monitoring for Adherence for Heart Failure (REMADHE)	1. A significant association between spirituality and medication adherence was reported (*r* = 0.26, *p* = 0.003) 2. Intrinsic religiosity showed association with medication adherence score (*r* = 0.20, *p* = 0.02)	1. Cross-sectional design was used; it was inadequate to show a causal relationship 2. Future studies need to determine whether these findings are replicable in different religious and cultural backgrounds 3. The effect of spirituality on adherence to various heart failure management facets was not individually assessed	Spirituality has an important role in managing heart failure patients. This study suggests that all health care providers and the patients with heart failure should have awareness of the role of spirituality in medication adherence management
Kretchy I et al. (2013), Ghana (Kretchy et al., 2013)	Cross-sectional study (n = 400)	Hypertension, two tertiary hospitals in Ghana (KATH, KBTH)	1. Duke University Religion Index (DUREI) 2. Spiritual Perspective Scale (Christians 90%, Muslims 5%, Traditional religion 1%)	Morisky Medication Adherence Scale (8-item)	93.3% of the patients were poorly adherent to their medications. Spirituality (*p* = 0.018), but not religiosity (*p* = 0.474), was related directly to medication non-adherence	Generalization to a larger population is limited because they recruited the participants from tertiary hospitals only, while there are many patients with hypertension in other settings in Ghana	The high level of religiosity and spirituality of patients with hypertension increased their trust in divine healing instead of adhering to their medications
Greer DB & Abel WM (2017), USA (Greer & Abel, 2017)	Mixed-methods design (*n* = 20)	Hypertension, African American Baptist church in rural East Texas	The Brief Religious/Spiritual Coping Scale (Christianity)	Hill-Bone Compliance to High Blood Pressure Therapy scale	The dominant theme that emerged from participants interviews was prayer. Prayer helped the women adhere to their anti-hypertensive treatment regimen	1. Generalization to a larger population and inferential statistics were limited due to participants' very small sample size and heterogeneity 2. Data saturation was obtained with 20 participants, and the qualitative phase of the study was weighted more than were the quantitative measures 3. Sampling was done in only one rural area in Texas with only African American women; generalizations to the larger population remain limited	This study suggests the importance of including R/S in the management of hypertension. Furthermore, prayer helps patients take their medication regularly
Park CL et al. (2008), USA (Park et al., 2008)	Cross-sectional study (*n* = 202)	Congestive heart failure (CHF), Cincinnati Veterans Administration Medical Center and University of Cincinnati Medical Center	Four subscales from the NIA/Fetzer Brief Measure of Religion and Spirituality (BMMRS) (No religion reported)	Compliance measure developed by Sherbourne, Hays, Ordway, DiMatteo, & Kravitz (1992)	1. Religious commitment was predictive of more adherence to CHF-specific behaviors (reporting new symptoms, exercise, medication, and stress management)	1. A majority of the participants were men 2. The assessment of adherence behaviors was based on self-report 3. The study should be considered more exploratory than definitive, focusing on a single disease and a single group of individuals 4. Other variables that may account for the relationships between religion and health, such as personality, were not assessed	These results suggest that some aspects of religiousness have positive effects, other aspects have adverse effects, and still, others appear unrelated to the performance of particular health behaviors
Yon AS (2013), USA (Yon, 2013)	Cross-sectional study-pilot study (*n* = 62)	Hypertension, Chatham Crossing Medical Center	1. Duke University Religion Index (DUREI) 2. Spiritual Health Locus of Control Scale (SHLCS)/Self-ranking of spirituality (Christianity)	1. Morisky Compliance Assessment Scale (MMAS 4-Item) 2. Morisky Medication Adherence Scale (MMAS 8-item)	1. A positive relationship with adherence was observed for organized religiosity (OR = 1.79; 95% CI 0.58–5.46), non-organized religiosity (OR = 1.05; 95% CI 0.37–2.99), and for intrinsic religiosity (OR = 1.41; 95% CI0.46–4.34), where patients with high religiosity had higher odds of reporting adherence than those with low religiosity 2. After controlling for race, self-ranking of spirituality was significant in relation to self-reported adherence (*p* = 0.04) 3. Patients who rated themselves as highly spiritual had higher odds of reporting adherence to their medications than had those with low spirituality (OR = 4.12; 95% CI 1.09–15.61)	1. The results may not be generalizable to other chronic diseases, younger adults, or populations outside the geographical region of Central North Carolina 2. The cross-sectional design of the present study did not permit determinations of causal relationships among the variables 3. The unobserved heterogeneity or unmeasured confounding affected the findings (as other mediating variables affect the relationships between spirituality, adherence behavior, and blood pressure) 4. The current study may have been heavily biased toward the Christian faith due to its population and the geographical region in which it was conducted	A better understanding of the mechanisms and role of spirituality in medication-taking behavior and health outcomes will aid researchers and health professionals develop culturally sensitive and patient-centered interventions to improve medication adherence and cardiovascular outcomes
Harvin LA (2018), USA (Harvin, 2018)	Quasi- experimental design (*n* = 10)	Hypertension, Central Church of Christ	Spiritual Perspective Scale (SPS) (Christianity)	The medication adherence section is composed of three questions regarding medication usage	The Wilcoxon test revealed a statistically significant increase in medication adherence scores of the participants following the hypertension management sessions,(*p* = 0.034)	1. Convenience sampling was employed in the selection of participants 2. Generalization to a larger population is limited due to the small sample size	Results of this project contribute to the limited body of knowledge regarding spirituality and its potential role in managing chronic diseases, especially among African Americans
Loustalot F (2006), USA (Loustalot, 2006)	Cross-sectional design (*n* = 5302)	Hypertension (home, office, or Jackson Heart Study (JHS))	4 measures utilized to assess religion and spirituality including organizational religiosity (OR), non-organizational religiosity (NOR), religious coping (RC), and daily spiritual experiences (DSE) (No religion reported)	31 of the JHS Medication Survey Forms	1. Those with more religious activities were more likely to be categorized as hypertensive 2. Organized religious had a negative nonsignificant relationship with hypertensive (*B* = -0.059, Odds Ratio = 0.943, *p* = 0.566)	1. Instrumentation, sample, and research design. The inclusion of religious media as a component of the OR measure made it difficult to assess the contribution of formal attendance in religious activities versus more-private watching of televised church activities 2. The data utilized for this study were from the baseline exam of the JHS. As these are the only current data available, the study is cross-sectional and is unable to speak to fluctuations among variables	This study supports the potential buffering effect of religion and spirituality on hypertension with lower levels of actual blood pressure among those reporting more religious or spiritual practices
Black G et al. (2006), USA (Black et al., 2006)	Cohort study design (*n* = 95)	Heart failure, Outpatient heart failure clinic and inpatient units	Spirituality Assessment Scale (SAS) (Christianity)	Heart Failure Compliance Questionnaire (HFCQ)	There was no relationship or correlation between the variables of spirituality and compliance among the Heart Failure participants (*r* = 16,393; *P* = 0.115)	1. Small sample size 2. The reliability of the HFCQR 3. The degree of compliance can change from 1 day to the next, and even hour by hour for these patients 4. Situational factors such as illness, family gatherings, family illness, and the business of holidays may affect compliance 5. The use of self-reports, convenience sampling, and response rate bias	The findings of this study may have practical implications for health care professionals helping individuals cope with a chronic illness

#### Measures of Religiosity/Spirituality

A variety of R/S measures were utilized in these studies, including a measure of nine R/S questions that were developed by the investigators (Abel & Greer, 2017): organizational religious activity (ORA); non-organizational religious activity (NORA); intrinsic religiosity (IR) (Alvarez et al., 2016; Kretchy et al., 2013; Yon, 2013); positive and negative R/S coping (Greer & Abel, 2017); R/S commitment (Park et al., 2008); active and passive spiritual health locus of control beliefs (Yon, 2013); spiritual views, the extent to which these views were held, and engagement in spiritually related behaviors (Harvin, 2018); and purpose and meaning in life, inner resources, interconnectedness, and transcendence (Black et al., 2006). Different types of instruments were used to assess R/S. Two measures were used in more than one study: the Duke University Religion Index (DUREL) (Alvarez et al., 2016; Kretchy et al., 2013; Yon, 2013) and the Spiritual Perspective Scale (SPS) (Harvin, 2018; Kretchy et al., 2013). A detailed description of the tools and scoring systems that were used is presented in Table 2.

**Table 2:** Religiosity and spirituality measures used in the included studies

Religiosity and/or spirituality measure (Reference)	Number of items	Instrument description	Scoring system
Nine spiritual/religious questions developed by the investigator (Abel & Greer, 2017)	9	Questions were reviewed for face validity by three community-dwelling African American women and for content validity by two nurse scientists with spiritual/religious training. Cronbach’s alpha was run on the four Likert-type questions, yielding an acceptable coefficient for newly developed items of 0.524	NA
1. Duke University Religion Index (DUREL) (Alvarez et al., 2016)	5	The DUREL scale has five items that describe three dimensions of religiosity known to best correlate with health-related outcomes: organizational (ORA); non-organizational (NORA); and intrinsic religiosity (IR)	The score ranges from 1 to 30 points, and higher scores indicate elevated levels of religiosity
2. The World Health Organization Quality of Life Spirituality, Religiosity and Personal Beliefs (WHOQoL-SRPB) (Alvarez et al., 2016)	32	It is composed of 32 items distributed in eight factors (Spiritual Connection, Meaning of Life, Awe & Wonder, Wholeness & Integration, Spiritual Strength, Inner Peace, Hope & Optimism and Faith) in a general index composed of 4 items (SRPB Global), originally of the SRPB domain of the WHOQOL-100	NA
1. Duke University Religion Index (DUREL) (Kretchy et al., 2013)	2	A two-item measure assessing two domains of religiosity: organized religious activity (ORA), i.e., (“How often do you attend church or religious meetings?”), and non-organized religious activity (NORA), i.e., (“How often do you spend time in private religious activities, such as prayer meditation, or Bible study?”). Responses range from 1 (“more than once a week”) to 6 (“never”) for ORA and 1 (“more than once a day”) to 6 (“rarely or never”) for NORA. DUREL has been validated in health research with Cronbach’s alpha values ranging from 0.75 to 0.88	Scoring is based on a separate regression model for each item
2. Spiritual Perspective Scale (SPS) (Kretchy et al., 2013)	10	(SPS) is designed to measure perceptions of the extent to which participants hold certain spiritual views and engage in spiritually related interactions. Each of the 10 items uses a 6-point Likert-type scale ranging from strongly disagree to strongly agree and is scored using the mean	Scores above the mean indicate high spiritual involvement, and those below the mean value indicate the reverse
Brief Religious/Spiritual Coping scale (Greer & Abel, 2017)	10	The brief RCOPE consisted of a 10-item, 4-point Likert scale. Five positive items addressed searching for a spiritual connection, collaborative religious coping, seeking spiritual support, benevolent religious reappraisal, and ritual purification, while the five negative items addressed punishing God reappraisal, spiritual discontent, self-directed religious coping, religious doubts, and anger at God. Cronbach’s alpha was 0.63 for the positive subscale, and 0.58 for the negative subscale	The positive and negative brief RCOPE subscale scores ranged from 1 to 4 (1 A great deal; 2 Quite a bit; 3 Somewhat, and 4 Not at all). The overall brief RCOPE item responses include 1 = Very involved, 2 = Somewhat involved, 3 = Not very involved, and 4 = Not involved at all. Scoring consists of summing positive and negative items. Positive and negative brief RCOPE subscale scores range from 5 to 20, with lower scores indicating higher levels of R/S coping
Religiousness was assessed with four subscales from the NIA/Fetzer Brief Measure of Religion and Spirituality (Park et al., 2008)	2	Religious support was assessed with two items regarding the extent to which one’s congregation would help with illness or other problems rated on a scale from 1 (none) to 4 (a great deal) (internal consistency reliability = .87). Commitment was assessed by asking participants the degree to which they tried to bring their religious beliefs over into other aspects of their lives from 1 (strongly disagree) to 4 (strongly agree). Positive and negative religious coping was each assessed with two items (e.g., “I work with God as partners,” and “I feel God is punishing me,” respectively) (internal consistency reliabilities = .82 and .89, respectively)	Items were rated on a scale from 1 (not at all) to 4 (a great deal)
The Duke University Religion Index (DUREL) (Yon, 2013)	5	Is a widely used five-item measure. The five single items measure organizational and non-organizational religiosity; the three-item subscale measures intrinsic religiosity. The overall scale has been shown to have high test–retest reliability (intra-class correlation = 0.91), high internal consistency (Cronbach’s α = 0.78–0.91) and high convergent validity with other measures of religiosity (*r*’s = 0.71–0.86)	Items in the subscales are scored on a five-to-six-point Likert-type scale
Spiritual Health Locus of Control Scale (Yon, 2013)	13	Is a 13-item, two-dimensional scale that assesses active and passive spiritual health locus of control beliefs. It was adopted from Holt and colleagues (2003, 2007). On the SHLCS, Cronbach’s α for the active dimension (11 items) ranges from 0.78 to 0.89 and from 0.56 to 0.76 for the passive dimension (2 items)	Item responses are scored on a four-point Likert-type scale that ranges from strongly disagree (1) to strongly agree (4)
Self-Ranking of Spirituality (Yon, 2013)	1	Is a one-item assessment of spiritual intensity that asks participants to rate the extent to which they consider themselves spiritual/religious. This item was adopted from the brief multidimensional measure of religiousness/spirituality (BMMRS) developed by a panel of experts on spirituality and health research at the Fetzer Institute and the National Institute on Aging and has been included on numerous spiritual surveys in combination with other spiritual measures; it has also been used as a single-item measure of spirituality	Responses range from 4 (very spiritual/religious) to 1 (not spiritual/religious et al.)
The Spiritual Perspective Scale (SPS) (Harvin, 2018)	10	Is a 10-item questionnaire that measured participants’ spiritual views and the extent to which they hold those views and engage in spiritually related behaviors. Examples of questions that inquire about frequency of spiritually related behaviors include “How often do you engage in private prayer or meditation?” and “I seek spiritual guidance in making decisions in my everyday life. Positive correlations between the scale and spiritual backgrounds have been noted, with all item-scale correlation’s above 0.60 and the Cronbach’s alpha above 0.90	Responses can range from 1 to 6 on a Likert-type scale, 1 = Not Likely to 6 = Always. Respondents scores were averaged to arrive at a spiritual perspective score, which can range from 1.0 to 6.0
The 4 measures utilized to assess religion and spirituality included organizational religiosity (OR), non-organizational religiosity (NOR), religious coping (RC), and daily spiritual experiences (DSE) (Loustalot, 2006)	5	All measures were obtained as part of the self-administered Approach to Life C form completed by the participant following the HII and returned to the clinic at the time of the baseline exam. Each measure was assessed as a continuous variable. OR, a measure of organized religious activities including attendance at religious services, was measured in question 1. The question states, “In general, how often do you attend the main worship service of your church or otherwise participate in organizational religion (such as watching services on TV, listening to services on the radio, participating in Bible study groups, etc.)?” NOR, the private practice of religious activities outside of the church, synagogue, or other place of worship was assessed in question 2. It states, “Within your religious or spiritual tradition, how often do you pray privately or meditate in places other than at church, mosque, temple, or synagogue?” Both OR and NOR have limited publications founding their psychometric properties, but are frequently used in social surveys and health-related research	OR and NOR were measured using an 8-level Likert type scale, ranging from “more than once a day” to “never.” Lower scores were correlated with more religious activities
Spirituality Assessment Scale (SAS) (Black et al., 2006)	28	The SAS comprises 28 questions with a 5-point Likert scale and is divided into four subscales: (1) purpose and meaning in life, (2) inner resources, (3) unifying interconnectedness, and (4) transcendence. The SAS has demonstrated high internal consistency and reliability (α = 0.92). Reliability of the SAS in the current study was 0.89. Internal consistency of SAS subscales for purpose and meaning in life, inner resources, unifying interconnectedness, and transcendence were 0.746, 0.732, 0.731, and 0.64, respectively	Scores range from 28 to 140, with a lower score indicating a higher level of spirituality

#### Measures of Medication Adherence

The tools used to measure medication adherence varied. These included the Repetitive Education and Monitoring for Adherence for Heart Failure (REMADHE) (Alvarez et al., 2016), adherence tools developed by the investigators (Harvin, 2018; Park et al., 2008), the JHS Medication Survey Forms (Loustalot, 2006), and the Heart Failure Compliance Questionnaire (HFCQ) (Black et al., 2006). Additionally, the 14-item Hill-Bone Compliance Scale was used in two studies (Abel & Greer, 2017; Greer & Abel, 2017), and the 4- and 8-item Morisky Medication Adherence Scale was used in two studies (Kretchy et al., 2013; Yon, 2013). A detailed description of each adherence instrument is presented in Table 3.

**Table 3:** Medication adherence measures used in the included studies

Medication adherence measure (references)	Number of items	Instrument description	Scoring system
14-item Hill-Bone Compliance to High Blood Pressure Therapy scale (W. M. Abel & D. B. Greer, 2017)	8	The Medication Adherence (eight items) subscale, along with one item that addressed prescription refills	Responses are scored on a 4-point Likert scale from 1 (“none of the time”) to 4 (“all of the time”). Lower scores represent greater adherence and can range from 9 (perfect adherence) to 36 (complete non-adherence)
Repetitive Education and Monitoring for Adherence for Heart Failure (REMADHE) (Alvarez et al., 2016)	10	The questionnaire is composed of ten questions involving four domains: use of medications (one question); food and fluids (seven questions); alcohol consumption (one question); and medical appointments (one question)	The score ranges between 0 and 26 points, with higher scores indicating better patient adherence. A REMADHE score equal to or higher than 18 points indicates adequate level of adherence
Morisky Medication Adherence Scale (MMAS) (Kretchy et al., 2013)	8	The scale has 8 items on which respondents score from zero to eight. The MMAS reliability measure was 0.83 for a study on hypertensive outpatients	The scale has 8 items on which respondents score from zero to eight and enables categorization as low adherence (< 6), medium adherence (6 – < 8), and high adherence (8). Patients who scored low and moderate were grouped as poorly adherent to allow for statistical analysis
Hill-Bone Compliance to High Blood Pressure Therapy scale (Greer & Abel, 2017)	14	The Hill Bone CHBPT scale is a 14-item 4-point Likert scale that was designed to assess compliance to blood pressure treatment and addresses three important domains: sodium intake, keeping appointments, and medication adherence. For this study, the term compliance was interchanged with the term adherence. Cronbach’s alpha for this sample of women was 0.81	The instrument is scored by summing the three subscales for a total score. Scores for each item range from 1 to 4 (1 = none of the time, 2 = some of the time, 3 = most of the time, and 4 = all of the time), with a total score range from 14 (minimum) to 56 (maximum). Higher scores indicate a lower level of adherence
Compliance measure developed by Sherbourne, Hays, Ordway, DiMatteo, & Kravitz (1992) (Park et al., 2008)	8	Eight health behaviors that are particularly important for CHF patients to adhere to (reporting new symptoms, exercise, stress management, medication, diet salt reduction, fluid intake, smoking, and alcohol use). Four items were loaded on a factor that named CHF-related behaviors, which included reporting new symptoms, exercising, taking medication, and managing stress. Factor loadings for the four items ranged from 0.472 to 0.772. The second factor included two behaviors involving diet (salt and fluid intake); factor loadings were 0.802 and 0.850. The third factor included two behaviors involving substance use (smoking and alcohol); factor loadings were .806 and .595. There was minimal cross-loading of items, and the overall variance accounted for by the three factors was 60.4 percent. The alpha coefficients for these three adherence factors were 0.65, 0.69, and 0.62, respectively	Participants were asked the following: 1. To what extent has your doctor discussed each of the following topics with you? Responses were rated on a scale from 1 (not at all) to 4 (to a great extent); 2. What has your doctor told you about each of the following? Responses were rated from 1 (nothing at all) to 4 (a great deal of information); and 3. How much do you follow your doctor’s advice about each of the following? Responses were rated from 1 (not at all) to 3 (completely)
Morisky Compliance Assessment Scale (Yon, 2013)	4	Morisky is a four-item scale that asks, (1) Do you ever forget to take your medicine? (2) Are you careless at times about taking your medicine? (3) When you feel better, do you sometimes stop taking your medicine? and (4) Sometimes if you feel worse when you take the medicine, do you stop taking it? The Morisky scale has been shown to be reliable (Cronbach’s α = 0.61) and to demonstrate both concurrent and predictive validity, but it does not capture behavior for specific medications. This measure is a general assessment of adherence and is not disease or medication-specific. It was selected for its feasibility, considering that the data for Aim 1 were collected in the context of a larger study	Its score is calculated by assigning one point for each answer of “no” and zero points for each answer of “yes.” Patients answering “yes” to one or more questions are viewed as possibly having problems with medication adherence
8-item Morisky Medication Adherence Scale (MMAS-8) (Yon, 2013)	8	This self-reported adherence scale was developed from the 4-item Morisky instrument used in Aim 1 (the pilot study) and supplemented with additional items to better capture barriers to adherence behavior. The new scale was determined to have higher reliability than the 4-item scale (α = 0.83 vs. α = 0.61) after its original validation in a sample of 1367 hypertensive patients; it was chosen in lieu of the 4-item scale used in Aim 1 because of its better reliability and because it is a disease-specific measure of adherence	MMAS-8 scores, which range from 0 to 8, have previously been dichotomized into two levels of adherence: high (score = > 6) and low (score = < 6)
The medication adherence section is composed of three questions regarding medication usage (Harvin, 2018)	3	Participants rated themselves using a seven-point Likert scale on how often they take their prescribed blood pressure medications	The possible range for the responses to the three questions are 0–21. Participants who score a 21 are considered adherent to their medication regimen
Item 31 of the JHS Medication Survey Form (Loustalot, 2006)	31	Medication habits were assessed by a series of 11 questions on medication-taking behaviors included in item 31 of the JHS Medication Survey Form. The questions were adapted from ‘the National Survey of Black Americans’, and limited psychometric data are available. The content of the questions has been noted as barriers to medication adherence in other studies	Potential responses included, “reason indicated,” “not a reason,” and “don’t know.” Participants who reported “reason indicated” or “don’t know” were classified as non-adherent for the particular item. Each of the 11 items was summed for a composite score ranging from 0 to 11, with higher scores representing less medication adherence
Heart Failure Compliance Questionnaire (HFCQ) (Black et al., 2006)	8	The HFCQR is a self-administered 5-point Likert scale that consists of 8 questions on compliance to prescribed activities. The original Heart Failure Compliance Questionnaire (HFCQ) consisted of five questions on compliance. A panel of 6 experts established content validity of the HFCQ, and an internal consistency of 0.68 was obtained using Cronbach’s α. For this study, the HFCQ was revised to include 3 critical prescribed activities: daily weighing, checking for edema, and early reporting of increasing symptoms	Scores range from 8 to 40, with lower scores indicating higher levels of compliance

### Study Quality Assessments

The CCAT assessed the quality of the included studies, and the scores ranged from 75 to 95%. A few of the studies addressed the representativeness of their sample or the generalizability of their findings to a broader population. Most studies reported study objectives and ethical considerations. Percentiles were used to classify the included studies into low quality (less than 76.5%), moderate quality (76.5–88%), and high quality (above 88%). Only one study (Yon, 2013) fell in the high-quality category, while the other eight studies were categorized as moderate or low in quality (Table 4). The kappa test demonstrated high agreement between the two reviewers (κ = 0.85).

**Table 4:** Quality assessment of the included studies

Authors (Pub. year) country (reference)	Preliminaries	Introduction	Design	Sampling	Data collection	Ethical matters	Results	Discussion	Total%	Classification
Abel & Greer (2017), USA (Abel & Greer, 2017)	4	5	4	4	4	4	4	4	83	Moderate
Alvarez et al. (2016), Brazil (Alvarez et al., 2016)	4	5	4	3	4	5	4	4	83	Moderate
Kretchy et al. (2013), Ghana (Kretchy et al., 2013)	4	5	4	3	3	3	4	4	75	Low
Greer & Abel (2017), USA (Greer & Abel, 2017)	4	5	5	4	5	4	4	4	88	Moderate
Park et al. (2017), USA (Park et al., 2008)	4	5	3	3	3	4	3	5	75	Low
Yon (2013), USA (Yon, 2013)	4	5	5	5	5	5	4	5	95	High
Harvin (2018), USA (Harvin, 2018)	5	5	4	3	4	3	4	4	80	Moderate
Loustalot (2006), USA (Loustalot, 2006)	4	5	4	5	4	4	4	5	88	Moderate
Black et al. (2006), USA (Black et al., 2006)	4	5	3	3	4	4	4	4	78	Moderate

### Association Between Religiosity/Spirituality And Medication Adherence

*Hypertension*. Six studies discussed the relationship between R/S and medication adherence in patients with hypertension and highlighted the importance of addressing spiritual issues when managing hypertension to improve patients’ adherence to antihypertensive medications. Four studies (Abel & Greer, 2017; Kretchy et al., 2013; Loustalot, 2006; Yon, 2013) used a cross-sectional design, one study (Greer & Abel, 2017) used a mixed-methods design, and one study (Harvin, 2018) used a single-group pre–post-experimental design. Prayer was an important coping mechanism of religiosity and was positively associated with medication adherence among African American women with hypertension (Greer & Abel, 2017). A positive correlation was reported between three dimensions of religiosity (organized, non-organized, and intrinsic religiosity) and medication adherence. An experimental study examined faith-based educational interventions in patients with hypertension (Harvin, 2018). The study found that patients had a higher score for medication adherence after the intervention (*p* = 0.034). In contrast, there were nonsignificant correlations between medication adherence and attending church/religious services, praying, reading the Bible/religious material, and strength of spiritual beliefs in African American women (Abel & Greer, 2017). However, the data supported that medication adherence improved as spiritual and religious beliefs increased, although the relationship was insignificant.

*Heart Failure*. Three studies investigated the association between R/S and medication adherence in patients with heart failure. Two studies (Alvarez et al., 2016; Park et al., 2008) used a cross-sectional design; the third study (Black et al., 2006) utilized a cohort study design. The results of the cohort study reported that there was no relationship between the variables of spirituality and medication adherence of heart failure patients (Black et al., 2006). On the other hand, one study supported a significant association between spirituality, intrinsic religiosity and medication adherence (Park et al., 2008). The third study reported mixed findings, with some aspects of religiousness that were associated with better medication adherence, while other aspects were associated with worse medication adherence (Alvarez et al., 2016). In summary, there were mixed findings concerning whether R/S contributes positively or negatively to medication adherence.

## Discussion

This study, which summarized the literature on the relationship between R/S and medication adherence and described the nature and extent of the studies evaluating the relationship in patients with CVDs, arrived at several conclusions. First, a better understanding of the relationship between R/S and medication-taking behaviors will help to develop culturally sensitive, spiritually based, and patient-centered interventions to improve medication adherence. These findings will subsequently contribute to positive health outcomes among patients with CVDs. Second, there is evidence of a positive or negative influence of religious and spiritual elements on adherence to pharmacological therapy (Badanta-Romero et al., 2018). Third, the findings suggest a significant association (positive or negative, depending on age, sex, and other confounding factors) between R/S and medication adherence in patients with CVDs.

These findings primarily apply to patients suffering from either hypertension or heart failure, since these were the only CVDs where the association was examined. Six of the included studies were conducted among patients with hypertension in the USA, while three studies were conducted among patients with heart failure. A significant association was observed concerning medication adherence with the three dimensions of religiosity reported to have a positive correlation among patients with hypertension (Kobayashi et al., 2015), where highly religious patients demonstrated a high adherence rate. Similarly, prayer was also associated with a high level of adherence among hypertensive patients (although only based on qualitative findings) (Greer & Abel, 2017). However, there was no correlation between R/S and adherence among heart failure patients in one study (Black et al., 2006). On the other hand, two studies reported significantly better adherence among those who had a higher level of R/S (Alvarez et al., 2016; Park et al., 2008).

We believed that a higher level of R/S would be associated with higher medication adherence among patients with CVDs, which might ultimately lead to better health outcomes. A recent systematic review of the relationship between R/S and QOL in CVD patients supports this research question (Abu et al., 2018).

To better understand the association between R/S and medication adherence, we need to define and differentiate between the two constructs (i.e., R/S). According to the current review, most studies did not mention the definitions of R/S or the difference between the two constructs. Some studies measured religiosity alone, some measured spirituality, and others measured both R/S. Thus, more research needs to be conducted to better understand the concepts of R and S (see Table 5).

**Table 5:** The definition of religiosity/spirituality from the included studies

Authors (publication year), country (reference)	Type of measurement R/S	Definition of the construct(s)	The effect of R/S on medication adherence	Comment(s)
Abel & Greer (2017), USA (Abel & Greer, 2017)	Religious beliefs and spirituality	Not mentioned “Though often used interchangeably, spirituality and religion are separate but related concepts”	There was no relationship between religious beliefs/ spirituality and medication adherence	In this study there was no clear definition of religious beliefs and spirituality
Alvarez et al. (2016), Brazil (Alvarez et al., 2016)	Spirituality	Not mentioned	There is an clear association between spirituality and medication adherence among patients with heart failure “1. A significant association between spirituality and medication adherence was reported (*r* = 0.26, *p* = 0.003) 2. Intrinsic religiosity showed association with medication adherence score (*r* = 0.20, *p* = 0.02).”	In this study there is no any definition of spirituality
Kretchy et al. (2013), Ghana (Kretchy et al., 2013)	Spirituality & Religious belief	“Formerly, spirituality and religiosity were examined as a one-dimensional construct” “religion is generally inter-related with spirituality since the former provides a structured environment for spiritual exploration and practices in life and the two constructs have been conceptualized to influence the development of each other. For example, religious practices encourage spiritual growth and spiritual activities are often an important aspect of religious participation”	There is an relationship Between spirituality and medication adherence but not religiosity	The high level of religiosity and spirituality of patients with hypertension increased their trust in divine healing instead of adhering to their medications
Greer & Abel (2017), USA (Greer & Abel, 2017)	Religious/spirituality	Not mentioned	Prayer helped the women adhere to their anti-hypertensive treatment regimen	Health care provider should learn more about patient’s belief
Park CL et al. (2008), USA (Park et al., 2008)	Religion/Spirituality	Not mentioned	Religious commitment was predictive of more adherence to CHF-specific behaviors (reporting new symptoms, exercise, medication, and stress management)	few studies that have specifically documented some relationship between religiousness and adherence
Yon (2013), USA (Yon, 2013)	Spirituality	Not mentioned	The current study suggested that there is an association between spirituality and medication adherence among older patients with hypertension	The discovery of these associations provides direction for future studies that will aid in understanding how health professionals can use this information to provide culturally sensitive and patient-centered care that will improve medication adherence and cardiovascular outcomes
Harvin (2013), USA (Harvin, 2018)	Spirituality	Not mentioned	The Wilcoxon test revealed a statistically significant increase in medication adherence scores of the participants following the hypertension management sessions, (*p* = 0.034)	Results of this project contribute to the limited body of knowledge regarding spirituality and its potential role in managing chronic diseases, especially among African Americans
Loustalot (2006), USA (Loustalot, 2006)	Religion and spirituality	Religion and spirituality may appear to be used interchangeably throughout this text and, in many cases, the degree of overlap allows for little distinction. However, they were conceptually defined as separate concepts, each with individual and overlapping attributes. Religion was defined as rituals, practices, and experiences involving a search for the sacred (i.e., God, Allah, or other similar figure) that are shared within a group. Spirituality was defined as a search for meaning and purpose in life, which seeks to understand life’s ultimate questions in relation to the sacred. Where religion may be formal, organized, group-orientated, ritualistic, or objective, spirituality has been viewed as informal, non-organized, self-reflective, experiential, and subjective (Koenig et al., 2001; Loustalot, 2006)	1. Those with more religious activities were more likely to be categorized as hypertensive 2. Organized religious had a negative nonsignificant relationship with hypertensive (*B* = -0.059, Odds Ratio = 0.943, *p* = 0.566)	This study supports the potential buffering effect of religion and spirituality on hypertension with lower levels of actual blood pressure among those reporting more religious or spiritual practices
Black et al. (2006), USA (Black et al., 2006)	Spirituality	Spirituality bestows multiple positive psychologic and psychosocial benefits to persons with chronic illness. Increased optimism and hope, positive meaning in life, and positive self-image were defined as invaluable byproducts of spirituality by patients dying of heart failure. Psychosocially, an important characteristic of spirituality includes community support for heart failure patients. Morris found that a relationship existed between higher spirituality levels and disease regression in patients with coronary artery disease. Koenig indicated a correlation between spirituality and less need for acute care and long-term services. A study by Hardin et al. concluded that the inclination to use prayer and meditation was less prevalent in patients with later stages of heart failure than in patients experiencing earlier stages of the disease	There was no relationship or correlation between the variables of spirituality and compliance among the heart failure participants (*r* = 16,393; *P* = 0.115)	The findings of this study should be interpreted with caution and possibly as inconclusive due to the small sample size

As mentioned previously, medication adherence is vital to ensure better health outcomes. Movahedizadeh et al. (2019) found a significant positive correlation between religious beliefs and medication regimen adherence (Movahedizadeh et al., 2019). Religious beliefs and spirituality are social determinants that can increase patients’ abilities to manage their disease and encourage their recovery. Patients must believe that medication-taking behaviors are an effort toward improved health and wellness. Medicine is a means to improve health, and patients must have faith in the medicine. Therefore, religion impacts a person’s use of medication.

It is not necessary to assume that if a person has high spirituality, he or she will have high religiosity because religiosity depends on practice and commitment. “Religiosity is associated with organized worship, while spirituality is defined as the internalization of positive values” (Mattis, 2000). To assess the association between religiosity/spirituality and medication adherence, we first need to identify whether the patients believe in religiosity, spirituality or both and bring their religious beliefs and spiritual thoughts into all of their life situations (Zimmer et al., 2019). Zimmer and coworkers (2019) suggested defining the term ‘religiosity’ in the language as “a belief, which is complete obedience, being driven by a certain thought or doctrine, and walking with its passengers and in its paths. As for the concept of religiosity as a term, it is a set of principles and values, which is embraced by a community of beliefs, words, and deeds, and in many cases, these values can affect the details of our lives and decisions, including for health issues, while for the concept of spirituality, it “is concerned with the soul, values and way of thinking about the world.” Those who are religious can also be spiritual and vice versa. In summary, the difference between R and S depends on the population's cultural and religious backgrounds in addition to personal spiritual beliefs.

We found that several different tools were used to measure R/S. Most of the included studies used existing validated scales (Alvarez et al., 2016; Black et al., 2006; Greer & Abel, 2017; Harvin, 2018; Kretchy et al., 2013; Loustalot, 2006; Park et al., 2008; Yon, 2013); however, one study utilized an investigator-developed scale to measure R/S among African American women (Abel & Greer, 2017). The Duke University Religion Index (DUREL), which contains three dimensions of religiosity (intrinsic, organizational, and non-organizational religiosity), has been used in several studies (Koenig & Büssing, 2010).

## Study Strengths And Limitations

To our knowledge, this systematic review is the first to examine the relationship between R/S and medication adherence among patients with CVDs. We used international guidelines to conduct these systematic reviews and searched approximately seven electronic databases. This review will ultimately create more research opportunities to deliver practical, tailored interventions to improve patient medication adherence and health outcomes.

However, there were limitations to this review. First, we excluded non-English articles, which could lead to publication bias. Second, most of the included studies had small sample sizes and were conducted among patients with hypertension, affecting the generalizability of our findings. Third, most studies were conducted in the USA, which may not sufficiently represent all types of religions or religious affiliations. Fourth, most of the studies utilized subjective instead of objective measures of adherence. Fifth, the data retrieved in this systematic review were not meta-analyzed because of the high methodological heterogeneity with large variability between studies in terms of the study design, instruments used to assess R/S, definition of outcome measures, etc. We applied a narrative approach to data synthesis in this review. Finally, according to the CCAT, the evaluation of the included studies was deemed to be of low-to-moderate quality.

## Important Knowledge From The Current Review

This systematic review collected all significant evidence about the connection between religiosity, spirituality and medication adherence among patients with CVDs. In addition, it has been noticed that the problem of medication non-adherence is only seen among patients with chronic CVDs such as hypertension and heart failure. However, no study has been performed on other types of acute CVDs, such as coronary artery disease, angina, or myocardial infarction. Patients with acute CVDs are cautious about taking their medications as per health care professionals’ instructions due to disease severity and their critical condition. Hence, we found no studies that discussed the relationship between religiosity, spirituality and medication adherence among patients suffering from acute CVDs. The researchers believe that this is the reason for the dearth of literature discussing medication adherence and other acute CVDs. Our systematic review supported the results of a meta-analysis that was carried out to prove that religious involvement is associated with low mortality and improved illness (McCullough, Hoyt, Larson, Koenig, & Thoresen, 2000).

## Practical Implications And Recommendations

The current systematic review will help clinicians and researchers be mindful of the gap in the literature, particularly among patients with CVDs in areas where religion is particularly important, i.e., religious/cultural traditions may strongly influence compliance with medications. Furthermore, religion and spirituality could be psychosocial factors and provide biological benefits for recovering from physical and mental disorders. Therefore, professionals should be aware of the impact that R/S has on a patient's life and incorporate these beliefs in care planning to support a holistic patient care approach. In addition, it is necessary to design interventions to promote positive coping strategies.

Future studies need to examine R/S and medication adherence in patients suffering from myocardial infarction, arrhythmia, and various levels of coronary artery disease. These findings should assist in designing comprehensive and effective interdisciplinary interventions, i.e., education and counseling programs, to address the influences of R/S on the management of chronic diseases such as CVDs. Future research in this area is greatly needed, particularly prospective studies and randomized clinical trials including patients with a more diverse range of CVDs, larger samples, and patients from different geographical locations, cultures, and religious backgrounds. In addition, understanding the relationship between R/S and medication adherence might help design spiritually integrated interventions (e.g., spiritual counseling) to improve medication-taking behaviors and adherence in patients with CVDs.

## Conclusions

In summary, the current comprehensive review of the relationship between R/S and medication adherence among patients with CVDs was missing from the literature. A few studies have examined the relationship between religiosity/spirituality and medication adherence in patients with CVDs (hypertension and congestive heart failure). No studies investigating other types of CVDs were found. The studies reviewed had observational designs and found inconsistent relationships between R/S and medication adherence. The level of medication adherence among patients with CVDs (heart failure and hypertension) can be positively and negatively influenced by religious activity (prayer, intrinsic religiosity) and spirituality. Indeed, throughout history, religiosity and spirituality have been increasingly recognized as impacting health and treatment. Finally, understanding the relationship between religiosity/spirituality and medication adherence will help design educational programs and spiritually integrated interventions to improve medication-taking behaviors in patients with CVDs.

## Data Availability

Not applicable.
